# Climate change impacts on water sustainability of South African crop production

**DOI:** 10.1088/1748-9326/ac80cf

**Published:** 2022-07-25

**Authors:** Sara Bonetti, Edwin H Sutanudjaja, Tafadzwanashe Mabhaudhi, Rob Slotow, Carole Dalin

**Affiliations:** 1 Soil Physics and Land Management Group, Wageningen University and Research, Wageningen, The Netherlands; 2 Institute for Sustainable Resources, University College London, London, United Kingdom; 3 Department of Physical Geography, Faculty of Geosciences, Utrecht University, Utrecht, The Netherlands; 4 Centre for Transformative Agricultural and Food Systems, School of Agricultural, Earth and Environmental Sciences, University of KwaZulu-Natal, Pietermaritzburg, South Africa; 5 International Water Management Institute (IWMI-GH)—West Africa Regional Office, Accra, Ghana; 6 Centre for Transformative Agricultural and Food Systems, School of Life Sciences, University of KwaZulu-Natal, Pietermaritzburg, South Africa; 7 Department of Genetics, Evolution & Environment, University College London, London, United Kingdom

**Keywords:** water sustainability, water debt indicator, South African crop production, water resources, food security, water footprint, climate change

## Abstract

Agricultural production in arid and semi-arid regions is particularly vulnerable to climate change, which, combined with projected food requirements, makes the sustainable management of water resources critical to ensure national and global food security. Using South Africa as an example, we map the spatial distribution of water use by seventeen major crops under current and future climate scenarios, and assess their sustainability in terms of water resources, using the water debt repayment time indicator. We find high water debts, indicating unsustainable production, for potatoes, pulses, grapes, cotton, rice, and wheat due to irrigation in arid areas. Climate change scenarios suggest an intensification of such pressure on water resources, especially in regions already vulnerable, with a country-scale increase in irrigation demand of between 6.5% and 32% by 2090. Future land use planning and management should carefully consider the spatial distribution and local sustainability of crop water requirements to reduce water consumption in water risk hotspots and guarantee long-term food security.

## 1. Introduction

The increasing global demand for agricultural products is placing unprecedented pressure on water resources [1–4]. Such a pressing market demand, combined with the severe water scarcity that characterizes many of the world’s regions, poses a challenge to the simultaneous assurance of food security and sustainable water resources management [5], especially under the future growing population [6] and climate change projections [7]. Critical levels have been reached between water demand for crop production and water availability in many of the world’s regions and particularly in water-limited countries [6], making the identification of such unsustainable states essential to devise a strategy for water resource management in agriculture [5].

A notable example is South Africa, where the agricultural sector supports a significant portion of the national economy and contributes massively to rural development, while coping with a growing water crisis [8, 9]. Even if agriculture only accounts for about 3% of the total national gross domestic product [10], it assures food security for the country and plays a key role in job creation and employment, with approximately 8.5 million people (${\approx}$14% of the country’s population) directly or indirectly dependent on this sector for employment and income [10], including both commercial and subsistence farming. At a regional and global level, South Africa is also a major food producer and exporter, being one of the two main trading hubs of southern Africa together with Zimbabwe [11, 12]. Despite the crucial role of the South African agricultural sector in local, regional, and global economy, crop production is highly threatened by limited water resources. In fact, over 80% of South Africa may be classified as semi-arid to arid, with only 18% being dry sub-humid to sub-humid, thus limiting the potential for crop cultivation [13]. The mean annual rainfall varies from less than 100 mm yr^−1^ in the west to over 1500 mm yr^−1^ in the east, with an average of approximately 450 mm yr^−1^ [14, 15]. Only ${\approx}$12% of South Africa’s total surface area can be used for rainfed crops [8, 13], making commercial agriculture production heavily dependent on irrigation (which accounts for approximately 60% of the total water withdrawals [8, 13]). Such limited water resources may limit crop production and subsequently contribute to food insecurity—a condition that is likely to worsen in a climate change scenario. Current predictions suggest that some areas of South Africa will experience decreasing rainfall and increased frequency of extremes such as drought events [11, 14, 16, 17]. These changes are likely to propagate into reduced water availability and crop yields [11], not only hindering agricultural exports and associated foreign income, but also threatening food security especially in rural communities still depending on rainfed crops and relying on natural systems for their livelihoods [16, 18].

Given the key role of water resources management for crop production in South Africa, a large number of studies examined the water use related to the agricultural sector. A first water footprint assessment for South Africa [19] showed that crop production contributes about 75% of the total national water footprint with maize, fodder crops, sugarcane, wheat, and sunflower seed accounting for 83% of the crop water footprint. In this assessment, the authors further explored catchment-scale blue water scarcity [20], showing that all major South African river basins experience water scarcity for at least 2 months a year. Other works on crop water use and sustainability under current climatic and management conditions as well as future scenarios either focused on specific crops and/or locations [17, 21–24] or considered South Africa within broader regional and global analyses [11, 12, 20, 25, 26]. As such, a spatially distributed analysis of local crop water sustainability, accounting for current production and irrigation requirements as well as future climate change scenarios, is still missing. This is paramount to provide a comprehensive picture of crop-related water requirements and sustainability as well as devise pathways for optimal land use planning and management strategies at the relevant spatial scales, so as to reduce current and future pressures on water resources and inform ongoing discussions on land reform.

**Figure 1.**
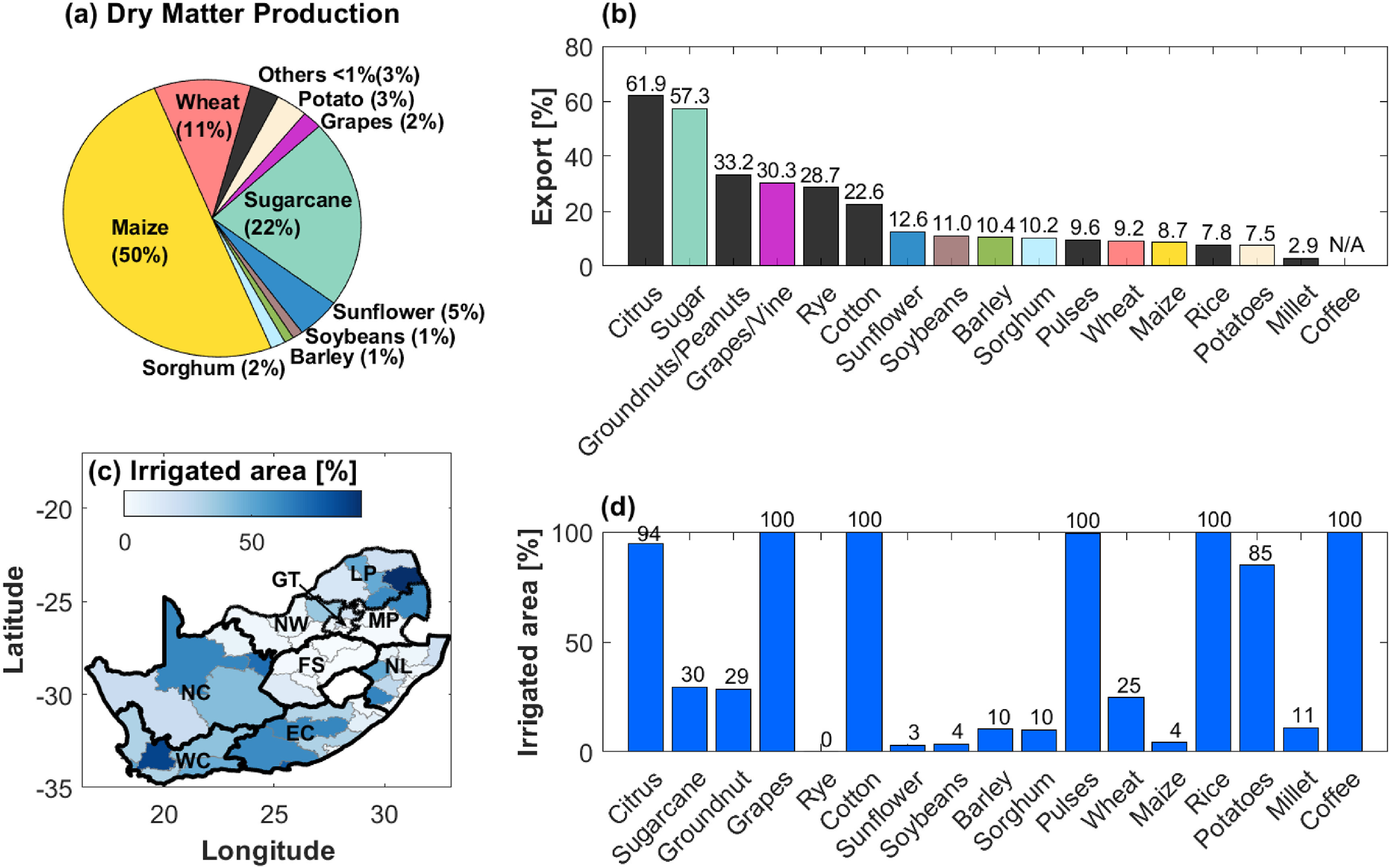
Crop production in South Africa (reference year 2000). (a) Percentage of total dry matter production of the 17 crops analyzed here (data from [28, 29]). The category ‘Others’ refers to crops with dry matter production $\lt$1% (i.e. citrus, groundnuts, rye, cotton, pulses, rice, millet, coffee). (b) Percentage share of production exported per each crop (color code is the same as in panel (a), data from FAOSTAT bilateral trade matrices were corrected for re-export following Dalin *et al* [3]). (c) Percentage of total harvested area that is irrigated (data from [28]), aggregated at the district level. Black and gray lines represent province and district boundaries, respectively. (d) Percentage of harvested area that is irrigated per crop type (data from [28]).

In this work, we evaluate the sustainability of water use for crop production, with a specific focus on South Africa. Specifically, the analysis is performed: (a) at fine spatial resolution (on a grid of 5 arc-min resolution, i.e. about 9 km), (b) by evaluating not only commonly used water requirement indicators, such as the water footprint (WF) and virtual water content (VWC), but also by assessing crop-specific blue water (i.e. surface water and groundwater resources) and green water (i.e. soil moisture) local sustainability in terms of water debt repayment time (WD), and (c) considering both current conditions and future climate change scenarios. As such, water sustainability is first evaluated, in a spatially distributed manner, for the 17 major crops produced in South Africa (figure 1) under current climatic and irrigation conditions (i.e. the reference year 2000). Evaluation of the WD indicator enables us to estimate the time necessary for the hydrologic cycle to renew the water used for annual crop productivity, thus providing a direct quantification of the local mismatch between water use and availability across different crop types, water sources, and production sites [26]. Specifically, the WD indicator identifies areas where the annual water footprint is unsustainable relative to the local resources, thus requiring strategic planning and management. We further evaluate the changes in water sustainability by crop and water sources—assuming current cropland location and crop production are maintained—under three climate change scenarios, which affect both crop water requirements and water availability. This analysis is performed by using, in each scenario, projections from five different climate models (i.e. GFDL-ESM2M, IPSL-CM5A-LR, HadGEM2-ES, MIROC-ESM-CHEM, NorESM1-M) of the Inter-Sectoral Impact Model Intercomparison Project (ISI-MIP) [27]. Comparing results based on this ensemble of models further enables us to investigate the uncertainty related to model estimates and projections. The analysis allows us to map the spatial distribution of source- and crop-specific water uses, detect the most unsustainable crops and hotspot regions for water risk (i.e. areas with local unsustainable crop water use), identify future trends under different levels of global warming, and ultimately provide guidelines for future interventions to improve sustainability of crop water use. Particularly, the ability to unfold the crop-specific responsibilities behind water resource overexploitation may be further employed in the analysis of virtual water flows associated to agricultural trades as well as to detect hotspot areas that may be deployed to different land uses (e.g. towards biodiversity conservation/restoration targets). Our findings, while specifically focusing on South Africa as a case study, provide general evidence for the need to differentiate the definition of water resource management strategies across different regions to account for local characteristics and offer valuable guidelines for other arid and semi-arid regions and similar environmental contexts (e.g. in southern Africa, South America, and Australia).

## 2. Methods

The root-zone soil moisture balance and the calculation of water requirements and sustainability indicators are performed at the cell level with a $5 \times 5$ arc-min resolution. We applied the methodology by Tuninetti *et al* [26, 30] and computed water sustainability using South African data for the year 2000 and future scenarios—definitions of WD are presented in appendix A while an overview of the methods for the calculation of the evapotranspiration over a growing season is provided in appendix B. Note that data sources used here are the same as in [26, 30], apart from meteorological forcings, some crop yield data, and renewability rates (see table S1 for details).

The study considers 17 crop groups (as defined by Portmann *et al* [28]): wheat, maize, barley, millet, sorghum, rye, rice, soy, sunflower, potato, sugarcane, groundnut, pulses, citrus, grapes, cotton, and coffee (see figure 1). Note that, in this classification, for some crop groups more than one primary FAO crop class was summed: maize (three FAO classes: maize, maize forage, green corn), rye (two FAO classes: rye, rye forage), sorghum (two FAO classes: sorghum, sorghum forage), citrus (five FAO classes: tangerines and mandarines, oranges, lemons and limes, grapefruit and pomelos, citrus fruit nes), pulses (11 FAO classes: bambara beans, dry beans, dry broad beans, chick peas, dry cow peas, lentils, lupins, dry peas, pigeon peas, vetches, pulses nes). Water sustainability indicators are computed for the reference year 2000 (i.e. the most referenced year in spatially distributed agricultural datesets available) and for future scenarios (every 10 years from 2010 to 2090). For future projections, we considered three Representative Concentration Pathways (RCP) scenarios (i.e. 2.6, 4.5, and 8.5), corresponding to different global warming trajectories. In these scenarios, we assumed that both production (in terms of crop harvested area, location, and crop yield) and irrigation (in terms of amounts and spatial distribution of irrigated areas) remain constant (equal to the reference year 2000), so as to analyze how the spatial distribution of water (un)sustainability may be affected by different levels of global warming.

Both for the reference case and for the future projections, we used climatological data (rainfall and reference evapotranspiration) and estimates of renewability rates from the PCR-GLOBWB 2 model [31], which were available based on five different climate models (GFDL-ESM2M, IPSL-CM5A-LR, HadGEM2-ES, MIROC-ESM-CHEM, NorESM1-M) from the ISI-MIP [27]. Long term monthly averages for the renewability rates were constructed from monthly estimates from the PCR-GLOBWB 2 model (taking a 10 year window centered around the year of interest), and annual renewability rates were then obtained by cumulating the monthly averages over the year. For the climatological data (rainfall and reference potential evapotraspiration), we used monthly averages considering a 3 year window centered around the year of interest. In the work here, we only distinguished between green (soil moisture) and blue (surface and groundwater bodies) water—we did not distinguish between surface and groundwater sources due to a lack of spatially distributed information on area equipped for irrigation with water from the different sources. All data sources used in this analysis as well as values of crop-specific parameters used in the calculations are provided in the Supplementary Information (tables S1 and S2).

## 3. Results

### 3.1. Water sustainability of South African crop production

The analysis of water sustainability of current crop production is first performed here for the reference year 2000. As detailed in appendices A and B, green and blue water requirements are evaluated by means of a soil water balance model and results are then contrasted with local renewability rates to evaluate crop- and source-specific water sustainability (i.e. WD calculation) at different spatial scales (from the grid cell to the district, province, and national levels). While the high resolution analysis can be used to trace back the causes of unsustainable water uses and identify site-specific interventions, aggregation at regional level is particularly useful to inform sustainability policies, especially in South Africa which is based on a distric-level economic development model.

**Figure 2.**
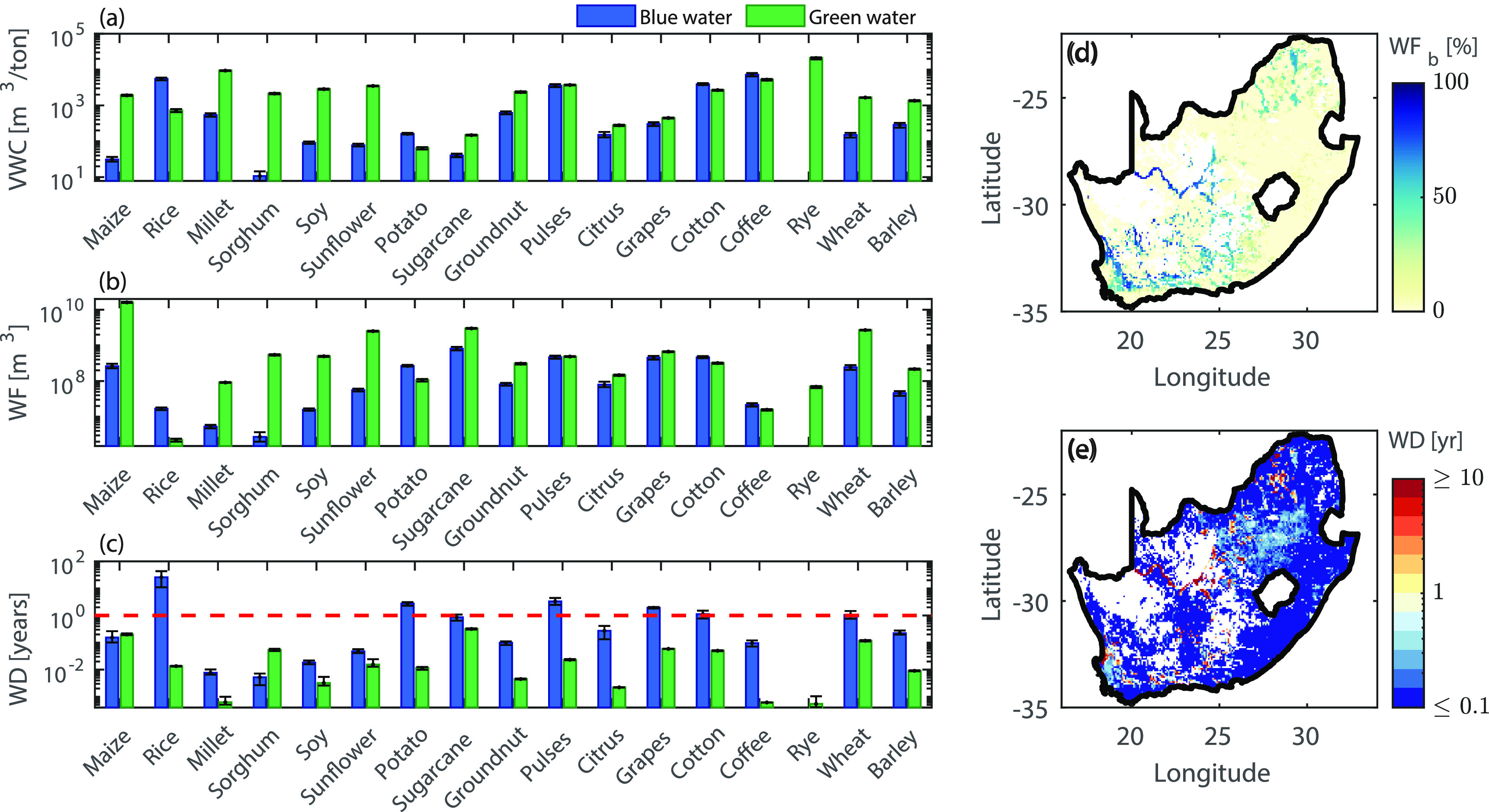
Water sustainability of current South African crop production. Country level values of green and blue (a) average VWC (i.e. production-weighted average), (b) total WF (i.e. sum over all cells), and (c) average WD (i.e. production-weighted average) for the reference year 2000. Box plots were obtained as averages from the five climate models, error bars show the maximum and minimum values obtained from the different climate models. The red dashed line in panel (c) marks the value of WD = 1 year. Spatial distribution of (d) percentage of blue WF and (e) WD values at the grid level (considering all crops, average across five climate models). The colorbar in panel (e) was truncated between 0.1 and 10 yr to favor comparison.

**Figure 3.**
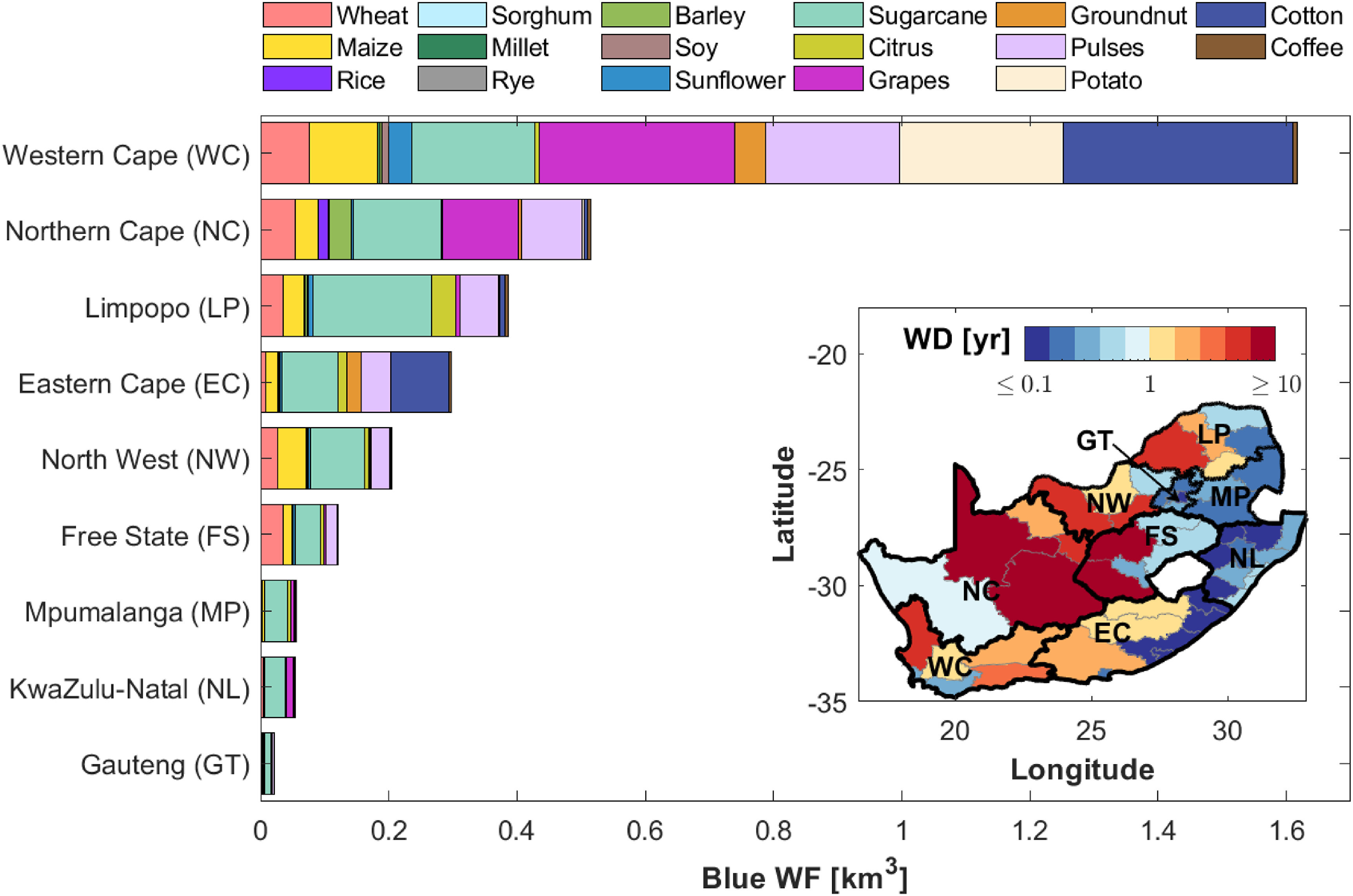
District and province-level water requirements. Crop-specific blue WF in South African provinces for the reference year 2000. The inset shows the district-level WD (black and gray lines represent province and district boundaries, respectively)—the colorbar was truncated between 0.1 and 10 yr to favor comparison.

Production of the 17 considered crops requires approximately 30.09 km^3^ of water per year, 10.5% of which is irrigation from surface water bodies and groundwater (i.e. ‘blue water’), the remaining 89.5% being from soil moisture (i.e. ‘green water’) (see table S3). Blue and green country-level VWC (volume of water necessary to produce a metric ton of good), WF (total volume of fresh water used for crop production), and WD (the ratio between the annual WF and the water annually available locally) for all the crops for the year 2000 are illustrated in figures 2(a)–(c) (see also figure S1). The green WF is dominated by maize (16 km^3^ yr^−1^), sugarcane (3 km^3^ yr^−1^), sunflower (2.5 km^3^ yr^−1^), and wheat (2.7 km^3^ yr^−1^)—in line with the analysis from Pahlow *et al* [19] based on data for the time period 1996–2005. The crops requiring the highest volumes of irrigation water are sugarcane (0.8 km^3^ yr^−1^), pulses (0.46 km^3^ yr^−1^), cotton (0.46 km^3^ yr^−1^), grapes (0.45 km^3^ yr^−1^), potato (0.26 km^3^ yr^−1^), maize (0.26 km^3^ yr^−1^), and wheat (0.24 km^3^ yr^−1^)—see figure 2(b) and table S3. Maize, wheat, sugarcane, cotton, and grapes were identified as dominant crops contributing to blue WF also by Pahlow *et al* [19]. Country-scale values of VWC show a relatively good agreement with those obtained by Pahlow *et al* [19] (see table S8). Such water requirements result in different water sustainability levels for each crop. Out of the 17 major crops analyzed, rice, potatoes, pulses and grapes were identified as water-unsustainable, as they consume more water than locally available (i.e. blue $\mathrm{WD}\gt1$ year, see figure 2(c) and table S3). Although some differences were observed in country-scale WD values across the different climate models (see error bars in figure 2(c)), this did not affect the water-sustainability/unsustainability classification of the various crops, apart from wheat and cotton (for which the blue WD oscillates around the critical value of 1 year depending on the climate model). The spatial distribution of total WD (i.e. maximum between green and blue WD) arising from all 17 crops is mapped in figure 2(e). High WD values are found in the Western and Northern Cape regions (most notably along the Orange river), revealing unsustainable production due to irrigation in arid areas (as confirmed by the spatial distribution of the percentage of water used for irrigation shown in figure 2(d)). Other vulnerable areas are found in the Limpopo, North West, Free State, and Eastern Cape provinces (figure 2(e) and inset of figure 3). Specifically, maximum values of district-level blue WD reach 8.5, 7.5, 26, 29.9, 2.5, and 6.8 years in some municipalities of the Limpopo, North West, Free State, Northern Cape, Eastern Cape, and Western Cape provinces, respectively, while district-level blue WD values are below the critical value of 1 year in all districts of Mpumalanga, Gauteng, and KwaZulu-Natal (see tables S4, S5 and inset of figure 3). Spatial patterns of crop water requirements are in good agreement with previous studies [19]. The observed spatial differences in WD levels across various production regions is the result of a combination of multiple factors, including the local pressure on water resources (e.g. due to low crop water use efficiency or intensive crop production), and the local ability of the hydrological cycle to support such pressure, embedded in the local renewability rates [26]. The observed spatially distributed values of WF and WD show different sensitivity to the choice of climate model. Specifically, the total WF is quite robust to the model choice, with variations of approximately ± 10% around the average (figures S2(k)–(o)). Similarly, the green WD was quite consistent across climate models, with the IPSL-CM5A-LR model providing the highest green WD values (up to ${\approx}$30% above average in certain areas) and the NorESM1-M model yielding the lowest green WD values (figures S2(a)–(e)). Conversely, the blue WD was highly sensitive to model choice (figures S2(f)–(j)), reflecting a high uncertainty in the surface and groundwater renewability rates across the climate models considered.

Lastly, figure 3 shows the contribution of each crop to the province-level blue WF. The major contributions to blue WF derive from the most irrigated crops, including grapes and pulses (especially in the Western and Northern Cape provinces), cotton (mostly in Western Cape and Eastern Cape), potatoes (primarily in Western Cape), and sugarcane (a major contributor to blue WF in all provinces—see also basin scale analysis in Pahlow *et al* [19]). Citrus fruits represent a major share of the blue WF only in the Limpopo region. Notably, maize and wheat, despite being two of the most produced crops (see figure 1(a)), represent a relatively small contribution to the total blue WF due to their mostly rainfed production (see figure 1(d)). We further note that a relatively large portion of the blue WF from sugarcane and grapes, which contribute to the local depletion of water resources and the observed high WD values, is related to internationally exported crops (figure 1(b)). This is particularly relevant in the context of evaluating the hidden environmental cost of crops produced for export as well as the responsibilities to reduce or bear such costs [32].

### 3.2. Crop water sustainability under future climate scenarios

To investigate the effects of future climate changes, water requirements and sustainability indicators are evaluated every ten years from 2010 to 2090 under different RCPs depicting a spectrum of possible climate policy outcomes and global warming trajectories, from the most stringent RCP 2.6, to the intermediate RCP 4.5, and the worst-case scenario of RCP 8.5. In these scenarios, we assumed that production (in terms of crop harvested area, location, and crop yield) remains constant (equal to the reference year 2000), so as to analyze how the spatial distribution of water (un)sustainability may be affected in the future if current production levels are sustained. Specifically, no assumptions are made on either future yields or the spatial distribution of crop production, in order to focus on climate change impacts on crop-specific water requirements and identify hotspot regions where those exceed projected water availability under a *status quo* scenario for agriculture.

**Figure 4.**
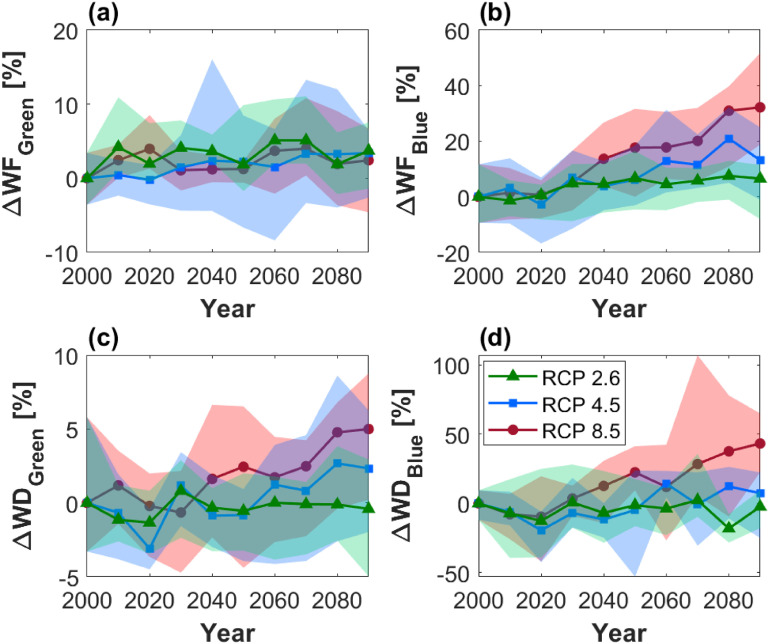
Crop water requirements and sustainability under future climate scenarios. Percentage variations (with respect to the reference year 2000) in country-level (a) green WF, (b) blue WF, (c) green WD, and (b) blue WD (considering all 17 crops). Green triangles, blue squares, and red circles refer to RCP 2.6, 4.5, and 8.5, respectively. The solid lines are the average from the five different climate models, while shaded areas mark the maximum/minimum values obtained from the different climate models (i.e. model uncertainty).

Country-level changes in WF and WD under climate change scenarios are shown in figure 4. While the green WF is expected to remain rather constant (figure 4(a)), a country-scale increase in water required for irrigation of between 6.5% and 32% (average across the five climate models) by 2090 is projected, depending on the RCP scenario (figure 4(b)), suggesting an intensified pressure on water resources. When contrasted with the predicted locally available water resources, this translates into an increase in country-scale blue WD values of 43% under the scenario with most severe climate change (RCP 8.5—figures 4(c) and (d))—under such scenario, despite the model uncertainty (shaded areas in figure 4), all climate models predict an increase in blue WD by 2090. Despite oscillations, WD values are expected to remain rather constant under RCP 2.6, while under RCP 4.5 an average increase of 2.3% and 9.5% is predicted for green and blue WD values, respectively (figures 4(c) and (d)). The spatial distribution of WD under future scenarios shows an increased unsustainability of irrigated crop production in some of the already most vulnerable provinces under RCP 8.5 (figures S5 and S6). Specifically, some districts in the Limpopo and Free State regions, which were classified as water-sustainable in the year 2000 analysis, are projected to become water-unsustainable by 2090 under the worst-case scenario (RCP 8.5), with fourfold increases in WD values (see figure S6, tables S6 and S7), mostly in relation to sugarcane, wheat, and pulses production.

**Figure 5.**
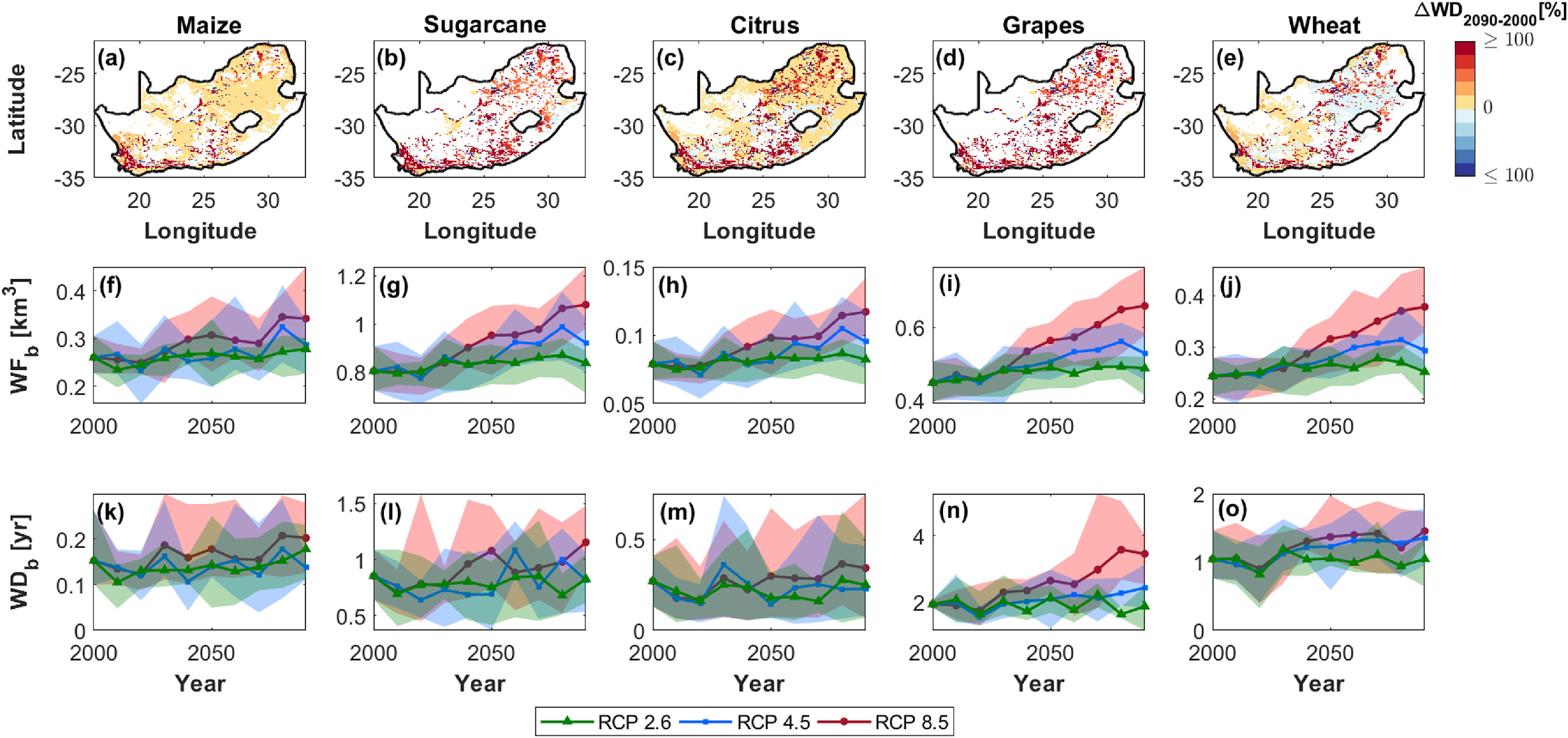
Crop-specific water sustainability under future climate scenarios. (a)–(e) Spatial distribution of percentage WD variations between year 2090 and year 2000 (RCP 8.5)—the colorbar was truncated between −100% and 100% to favor comparison. Time variations in country-level (f)–(j) blue WF and (k)–(o) blue WD. Green triangles, blue squares, and red circles refer to RCP 2.6, 4.5, and 8.5, respectively. The solid lines are the average from the five different climate models, while shaded areas mark the maximum/minimum values obtained from the different climate models. Results are shown for (a), (f), (k) maize, (b), (g), (l) sugarcane, (c), (h), (m) citrus, (d), (i), (n) grapes, and (e), (j), (o) wheat. Results for other crops are provided in the Supplementary Information.

When narrowing down the analysis to the different crops, maize, citrus, groundnut, coffee, barley, sorghum, soybean, millet, and sunflower are projected to remain water-sustainable (figures 5, S3 and S4). Conversely, the pressure on water resources of the already water-unsustainable crops (namely rice, potato, pulses, cotton, and grapes) is expected to either remain constant or increase, depending on the RCP scenario considered (figures 5, S3 and S4). Irrigated sugarcane production is likely to remain rather constant and water-sustainable, except under RCP 8.5 where it is expected to overshoot the WD = 1 year threshold (figure 5(l)). Wheat deserves special mention in the analysis here, as it represents one of the most important staple foods of the country and, although only a relatively small percentage of the total area planted is under irrigation (${\approx}25\%$, see figure 1(d)), irrigated wheat contributes approximately 30% of the national production [15]. According to our analysis, the sustainability of irrigated wheat production is borderline, with blue WD values oscillating around the threshold of 1 year between sustainable and unsustainable (figure 2(c)) and this WD is likely to either remain constant or increase up to approximately 1.5 years, depending on the RCP scenario considered (figure 5(o)). Increasing wheat production under irrigation is considered a viable option to improve national food security and reduce imports [15], but such changes in land management should be carefully planned by taking into account the spatial context and focusing on areas where current crop production is water-sustainable, in order to limit any additional pressure on water resources (see for example areas where blue WD is projected to decrease in figure 5(e)).

## 4. Discussion and conclusions

The analysis of water resources and depletion embedded in food production and global trade is paramount to delineate sustainable strategies for land and water resources management. Following Tuninetti *et al* [26], our work provides a framework to identify crop-specific water requirements and hotspots regions, for which holistic management of water resources is needed to concomitantly alleviate blue-water scarcity and guarantee food security, especially under future climate change. We note, however, that the assessment of crop water sustainability is performed here on a local basis, meaning that the amount of water necessary to produce certain crops is compared with the water locally available only—upstream flow and water transfers (as added water availability for agricultural use), and environmental flow requirements (as reduced water availability for agricultural use) are not considered in the analysis. Accounting for the availability of transferred water may be relevant for the water sustainability assessment in certain South African districts where water transfer schemes are in place (e.g. Vaalharts Irrigation Scheme, Orange-Fish Tunnel, and Tugela-Vaal Water Transfer Scheme). Neglecting water transfer schemes and upstream flow may lead to an overestimation of WD in downstream cells, however considering environmental flow requirements (i.e. removing these from blue water availability for irrigation) would further increase WD values. Despite such limitations, the approach here still allows one to locate the areas that are locally unsustainable, while not introducing additional uncertainty in WD related to environmental flow estimates available from the literature [26]. We further observe that crop production may suffer from the temporal mismatch between water use and availability throughout the year—a condition that is likely to worsen under projected climate change [14, 33]. While the WD indicator looks at this mismatch, it focuses on the potential overexploitation of annually renewable water resources. With regards to the analysis under future climate scenarios, in the work here we have investigated the effect of different global warming trajectories on the agricultural *status quo* without making any assumptions (and possibly introduce additional uncertainty) on future values and spatial distribution of crop yields, harvested areas, and areas equipped for irrigation. Future work should focus on the further assessment of water sustainability considering the effects of technological advancements (e.g. different seed varieties or irrigation technologies), market dynamics, food demands, and policies related to land reform and irrigated area expansion, which can impact projected production levels and management practices and have not been considered here.

The study reveals that the (un)sustainability of crop production in a certain region is the result of a delicate balance between crop types, water sources employed for production (i.e. rainfed or irrigated), and local climatology. Within this context, the promotion of drought-tolerant crops as well as underutilized indigenous crops (better adapted to prolonged periods of drought and characterized by good heat stress tolerance) may provide an important contribution to face the coupled challenges of food insecurity and water scarcity in hotspot regions, while also addressing the need for dietary diversity in rural communities [18, 34–36]. Furthermore, the identification of specific contributions of surface water and groundwater to the local resource availability—which was not possible in the present study due to a lack of spatially distributed data—might further help the identification of strategic water source areas [37] for crop production. This is particularly relevant not only in key food-producing regions of the world where excessive groundwater abstraction for irrigation is leading to rapid depletion of aquifers [3], but also in countries where groundwater resources still represent a relatively small contribution to the total water supply, such as South Africa (with about 3500 million m^3^ yr^−1^ of water from groundwater bodies estimated to be potentially available for further development [10]). Lastly, the ability to identify areas of water overexploitation for crop production may further inform land reform strategies, allowing the identification of regions to be devoted to different land uses (e.g. achieving Aichi biodiversity targets).

The work here quantifies current water stress issues in South Africa and assesses how these might worsen under different climate scenarios unless adequate adaptation measures are introduced. The delineation of such national and local scale water and agricultural strategies is a challenge that should consider spatially differentiated policies accounting for the local peculiarities of a region as opposed to ‘one-size-fits-all’ solutions.

## Data Availability

All data that support the findings of this study are included within the article (and any supplementary files).

## Appendix A. Water footprint, virtual water content, and water debt repayment time

Water sustainability is assessed by evaluating the water debt repayment time, $\mathrm{WD}_\mathrm{s,cr,l}$ (yr), defined as the ratio of the crop annual water footprint $\mathrm{WF}_\mathrm{s,cr,l}$ (m^3^) in the grid cell and the average renewable volume of water annually available in the grid cell [26],

\begin{eqnarray*} \mathrm{WD}_\mathrm{s,cr,l} = \frac{\mathrm{WF}_\mathrm{s,cr,l}}{A_\mathrm l \cdot R_\mathrm{s,l}} \end{eqnarray*}

where $R_\mathrm{s,l}$ (m yr^−1^) is the annual renewability rate of the water source, $A_\mathrm l$ (m^2^) is the cell area, and subscripts *s*, *cr*, and *l* refer to specific water sources, crops, and locations, respectively. WD has units of years and measures the time necessary for the hydrological cycle to renew the water used. When the annual WF is lower than (or equal to) the local water availability, (i.e. $\mathrm{WD} \leqslant 1$ year) the resource is sustainably used, meaning that the annual crop production only exploits the renewable portion of the local water resource. Conversely, when $\mathrm{WD} \gt 1$ year, the annual WF is unsustainable relative to the local resources, and crops are using water faster than the renewability rate, with a consumption of the locally available stocks or, in the case of surface water, a reliance on upstream sources.

Renewable soil moisture, surface water, and groundwater are derived from the PCR-GLOBWB 2 model [31] run with a *no use* setting which simulates the natural recharge rates of each water source. Specifically, renewable soil moisture ($R_\mathrm{sm,l}$) is the fraction of rainfall that infiltrates into the upper soil layer and is readily available for root water uptake and evapotranspiration. Renewable surface water ($R_\mathrm{sw,l}$) is the net cell runoff (i.e. the fraction of rainfall that flows to surface water bodies minus the evaporation from lakes and wetlands). Lastly, renewable groundwater ($R_\mathrm{gw,l}$) is the water that deeply percolates the soil layer and recharges the aquifer.

The total WD for a given source *s* and for all the crops cultivated in a grid cell *l* is equal to the sum of the water debts generated by each crop,

\begin{eqnarray*} \mathrm{WD}_\mathrm{s,l} = \sum_\mathrm{cr = 1}^{N_\mathrm{cr}} \mathrm{WD}_\mathrm{s,cr,l}, \end{eqnarray*}

where $N_\mathrm{cr}$ is the number of crops cultivated in the grid cell. Conversely, the total WD across the different sources is the maximum between the WD values of each source since all sources are simultaneously renewed over time. In the analysis here, we distinguished between green water (i.e. soil moisture, *s* = *g*) and blue water (i.e. surface water and groundwater bodies, *s* = *b*), so that the total $\mathrm{WD}$ is $\mathrm{WD}_\mathrm{cr,l} = \text{max}\left(\mathrm{WD}_\mathrm{b,cr,l},\mathrm{WD}_\mathrm{g,cr,l} \right)$. The regional scale value of WD for a certain crop and source is computed as a production-weighted mean,

\begin{eqnarray*} \mathrm{WD}_\mathrm{s,cr,\text{R}} = \frac{\sum_\mathrm{l \in \text{R}}\mathrm{WD}_\mathrm{s,cr,l} \cdot \mathrm{Pr}_\mathrm{cr,l}}{\sum_\mathrm{l \in \text{R}} \mathrm{Pr}_\mathrm{cr,l}}, \end{eqnarray*}

where *R* indicates the region of interest (e.g. country, province, district). Lastly, if all crops are considered together, the regional scale WD is obtained as a weighted mean (weighted by the water volume used by all crops in the cell, namely the cell water footprint, $\mathrm{WF}_\mathrm{s,l} = \sum_{N_\mathrm{cr}} \mathrm{WF}_\mathrm{cr,s,l}$),

\begin{eqnarray*} \mathrm{WD}_\mathrm{s,R} = \frac{\sum_\mathrm{l \in R}\mathrm{WD}_\mathrm{s,l} \cdot \mathrm{WF}_\mathrm{s,l}}{\sum_\mathrm{l \in R} \mathrm{WF}_\mathrm{s,l}}. \end{eqnarray*}

The $\mathrm{WF}_\mathrm{s,cr,l}$ is estimated as the product of the local crop virtual water content, $\mathrm{VWC}_\mathrm{s,cr,l}$ (m^3^ ton^−1^), and the annual crop production $\mathrm{Pr}_\mathrm{cr,l}$ (ton),

\begin{eqnarray*} \mathrm{WF}_\mathrm{s,cr,l} = \mathrm{VWC}_\mathrm{s,cr,l} \cdot \mathrm{Pr}_\mathrm{cr,l}. \end{eqnarray*}

The annual crop production is the product between the crop yields and harvested areas. Crop-specific harvested area (irrigated and rainfed) were taken from [28] while yield data were obtained from [29]. In regions yield data where not available, average crop-specific values based on year 2000 total planted area and production were used (see table S1 for details).

The $\mathrm{VWC}_\mathrm{s,cr,l}$ equals the water evapotranspired during the growing season in a year, $\mathrm{ET}_\mathrm{s,cr,l}$ (m^3^ ha^−1^), divided by the annual crop yield, $Y_\mathrm{cr,l}$ (ton ha^−1^),

\begin{eqnarray*} \mathrm{VWC}_\mathrm{s,cr,l} = \frac{\mathrm{ET}_\mathrm{s,cr,l}}{Y_\mathrm{cr,l}}. \end{eqnarray*}

Details about the computation of actual evapotranspiration per crop over the growing season are provided in appendix B. Lastly, in regions where more than one crop per year is planted and harvested, the actual evapotranspiration is calculated as a weighted average (with respect to the area cultivated during the growing period for each crop) of the actual evapotranspiration of each crop during their growing season,

\begin{eqnarray*} \mathrm{ET}_\mathrm{l} = \frac{\sum_\mathrm{cr}\mathrm{ET}_\mathrm{cr,LGP} \cdot A_\mathrm{cr}}{\sum_\mathrm{cr}A_\mathrm{cr}}, \end{eqnarray*}

where the total evapotranspiration per crop during the growing season is the sum of green and blue components.

## Appendix B. Crop evapotranspiration over a growing season

The crop water footprint is computed by means of a soil water balance model [30]. The total crop evapotranspired by a crop in a single growing season (LGP) is obtained by summing up the daily actual evapotranspiration over the growing season,

\begin{eqnarray*} \mathrm{ET}_\mathrm{cr,LGP} = \sum_{j = 1}^\mathrm{LGP}\mathrm{ET}_{\mathrm{cr},j}, \end{eqnarray*}

where the daily crop evapotranspiration is calculated as [38]

\begin{eqnarray*} \mathrm{ET}_{\mathrm{cr},j} = k_{\mathrm{c,cr},j} \cdot k_{\mathrm{s,cr},j} \cdot \mathrm{ET}_{0,j}, \end{eqnarray*}

$\mathrm{ET}_{0,j}$ being the daily potential evapotranspiration, *j* the day of the growing period, while $k_\mathrm{c}$ and $k_\mathrm s$ are the crop and water stress coefficients, respectively. The daily potential evapotranspiration is computed by linearly interpolating monthly averages and attributing the monthly values to the middle of each month (for sake of simplicity, we considered months 30 days long). The crop coefficient $k_\mathrm c$ varies during the growing season as

\begin{eqnarray*} k_{\mathrm{c},j} = \begin{cases} k_\mathrm{c,ini} &amp; \text{if } j \in \text{Stage I} \\[6pt] j \cdot \frac{k_\mathrm{c,mid}-k_\mathrm{c,ini}}{j-l_\mathrm I} &amp; \text{if } j \in \text{Stage II} \\[6pt] k_\mathrm{c,mid} &amp; \text{if } j \in \text{Stage III} \\[6pt] j \cdot \frac{k_\mathrm{c,end}-k_\mathrm{c,mid}}{j-l_\mathrm I-l_\mathrm{II}-l_\mathrm{III}} &amp; \text{if } j \in \text{Stage IV} \end{cases} \end{eqnarray*}

where the length of each stage ($l_\mathrm{st}$) is computed as a fraction ($p_\mathrm{st}$) of the length of the growing period. Values of the crop coefficients and length of the crop development stages were obtained from the literature [38, 39] (see tables S1 and S2 for details).

The water stress coefficient $k_\mathrm s$ varies between 0 (maximum water stress) and 1 (no water stress) and is evaluated considering rainfed and irrigated production separately. For the irrigated production the water stress coefficient is set to 1 (no stress) for the entire duration of the growing period, while for rainfed production it is evaluated as

\begin{eqnarray*} k_{\mathrm{s},j} = \frac{ \mathrm{TAW}_{j}-D_{\mathrm{mo},j}}{\mathrm{TAW}_{j}-\mathrm {RAW}_{j}} \end{eqnarray*}

where TAW (mm) is the total available water content in the root zone, RAW (mm) the readily available water content, and $D_\mathrm{mo}$ (mm) is the root zone depletion in the morning (i.e. water shortage relative to field capacity). $\mathrm{TAW}$ depends on the available water contents AWC (mm m^−1^) and the root depth $Z_{\mathrm{r},j}$ (m) as

\begin{eqnarray*} \mathrm{TAW}_{j} = \mathrm{AWC} \cdot Z_{\mathrm r,j}. \end{eqnarray*}

The available water content was taken from the Harmonized World Soil database v1.2, while maximum root depth ($Z_\mathrm{r,max})$ for each crop were obtained from [38]. The root depth was assumed to linearly increase during the first two growing stages and then maintained constant until harvesting. RAW is the water that crops use for evapotranspiration before water stress and stomata closure begins and is given by

\begin{eqnarray*} \mathrm{RAW}_{j} = \rho \cdot \mathrm{TAW}_j \end{eqnarray*}

where *ρ* is the depletion fraction coefficient (here assumed constant over the growing season). Values of *ρ* for each crop were obtained from [38] (see table S2). In rainfed production, the root zone depletion in the morning $D_{\mathrm{mo},j}$ is equal to the depletion recorded at the end of the previous day $D_{\mathrm{ev},j-1}$ minus the daily precipitation value *P*_*j*_,

\begin{eqnarray*} D_{\mathrm{mo},j} = D_{{\mathrm{ev},j-1}-P_j}. \end{eqnarray*}

Daily precipitation was obtained by uniformly distributing the monthly rainfall along the growing season with daily frequency. In the evening the root zone depletion decreases because of evapotranspiration as $D_{\mathrm{ev},j} = D_{\mathrm{mo},j}+\mathrm{ET}_{\mathrm{cr},j}$, with $\mathrm{ET}_{\mathrm{cr},j} = k_\mathrm c \cdot \frac{\mathrm{TAW}_{j}-D_{\mathrm{mo},j}}{\mathrm{TAW}_{j}-\mathrm{RAW}_{j}}\cdot \mathrm{ET}_{0,j}$. Note that losses by deep percolation are neglected and capillary rise is assumed equal to zero. Furthermore, water excess leading to negative $D_{\mathrm{mo},j}$ is cut off at zero (i.e. exceeding precipitation is assumed to be lost as surface runoff). In rainfed conditions, the water volume evapotranspired by the crop during the growth period is totally green (i.e. $\mathrm{ET}_\mathrm{g,LGP} = \mathrm{ET}_\mathrm{LGP}$). Conversely, in irrigated conditions, the daily volume of irrigation, *I*_*j*_, is determined assuming that the crop fully evapotranspires with no water stress as $I_j = D_{\mathrm{mo},j}-\mathrm{RAW}_j+k_{\mathrm{c},j}\mathrm{ET}_{0,j}$. In the evening, the root zone depletion is given by $D_{\mathrm{ev},j} = D_{\mathrm{mo},j}+\mathrm{ET}_{\mathrm{cr},j}-I_j$. Thus, the blue water corresponds to the irrigation water ($\mathrm{ET}_{\mathrm{b},j} = I_j$) while the green water is the difference between the total $\mathrm{ET}$ and $\mathrm{ET}_{\mathrm{b},j}$.

We note that, in the analysis here, no specific assumptions about irrigation methods were made—the right amount (not more, not less) of water needed to ensure no stress conditions is computed and considered as local irrigation requirement. For rice cultivation, we considered an additional volume per unit area of 200 mm, following the approach by [30] (see also [40]). This allows us to account for the water used to prepare the paddy fields by saturating the root zone in the month before sowing or transplanting [30].
